# Rapid neodymium release to marine waters from lithogenic sediments in the Amazon estuary

**DOI:** 10.1038/ncomms8592

**Published:** 2015-07-09

**Authors:** Tristan C. C. Rousseau, Jeroen E. Sonke, Jérôme Chmeleff, Pieter van Beek, Marc Souhaut, Geraldo Boaventura, Patrick Seyler, Catherine Jeandel

**Affiliations:** 1 GET, Université de Toulouse, CNRS, IRD, CNES, 14 Avenue Edouard Belin, Toulouse F-31400, France; 2 LEGOS, Université de Toulouse, CNRS, IRD, CNES, 14 Avenue Edouard Belin, Toulouse F-31400, France; 3 Universidade De Brasilia, UNB, LAGEQ, Campus universitário Darcy Ribeiro, Brasilia, DF 70.910-900, Brazil; 4 Hydrosciences Montpellier, Université de Montpellier-IRD-CNRS, Place Eugène Bataillon 34000, France

## Abstract

Rare earth element (REE) concentrations and neodymium isotopic composition (ɛNd) are tracers for ocean circulation and biogeochemistry. Although models suggest that REE release from lithogenic sediment in river discharge may dominate all other REE inputs to the oceans, the occurrence, mechanisms and magnitude of such a source are still debated. Here we present the first simultaneous observations of dissolved (<0.45 μm), colloidal and particulate REE and ɛNd in the Amazon estuary. A sharp drop in dissolved REE in the low-salinity zone is driven by coagulation of colloidal matter. At mid-salinities, total dissolved REE levels slightly increase, while ɛNd values are shifted from the dissolved Nd river endmember (−8.9) to values typical of river suspended matter (−10.6). Combining a Nd isotope mass balance with apparent radium isotope ages of estuarine waters suggests a rapid (3 weeks) and globally significant Nd release by dissolution of lithogenic suspended sediments.

## Abstract (web summary)

Neodymium isotopes are tracers for past and present ocean circulation and biogeochemistry. Here, the authors combine observations of neodymium and radium isotopes in the Amazon estuary and show that the rapid release of neodymium from river suspended sediments leaves a strong imprint on coastal sea water.

## Graphical abstract

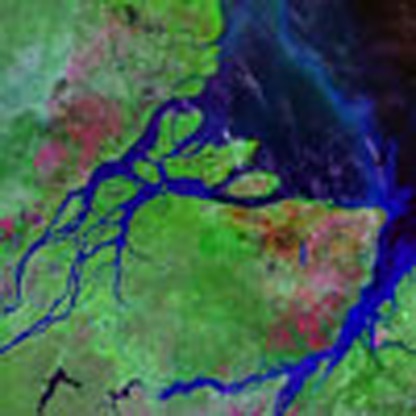

Understanding rare earth element (REE) speciation, dynamics and mass balance in natural waters is important because REE and neodymium (Nd) isotopes are used as tracers of biogeochemical processes, water mass transport and mixing in the modern ocean^1^,^2^,^3^,^4^ (and references therein) and as paleoproxies for past oceanic circulation patterns^5^,^6^ (and references therein). Dissolved and particulate river inputs of REEs to the oceans were recognized early on as being potentially important^7^,^8^,^9^. Later studies found the dissolution of continental atmospheric dust to be an additional REE source^10^,^11^,^12^,^13^. A marine Nd box model indicated, however, that to explain both global marine dissolved Nd concentrations and ɛNd, a large additional Nd source to the oceans was required^14^. The model study suggested that this source originated from the ocean margins and that it was on the order of 8,000 Mg per year. Using a general ocean circulation model that included the marine Nd cycle^15^, it was estimated that the release of 1–3% of the annual sedimentary Nd flux to continental margins, together with continuous dissolved/particle exchange with depth through the oceanic water column, are required to explain both the variations in global marine Nd concentrations and ɛNd. Two different Nd general ocean circulation models indicated the missing Nd flux to be on the order of 11,000 and 5,500 Mg per year 15,16. However, while the potential processes governing this sediment REE release have been discussed or hypothesized, their relative importance has not. These processes include estuarine transformations of the river solid discharge^17^,^18^,^19^,^20^, boundary exchange processes directly involving the sediments deposited on the margins and/or submarine groundwater discharge^21^,^22^,^23^,^24^,^25^.

Estuaries are important biogeochemical reactors where river fluxes of inorganic and organic matter influence the chemistry and biology of coastal waters and ultimately the open oceans. It is well-known that the flocculation of river-dissolved organic matter by sea salt drives the non-conservative behaviour of trace metals in estuaries^26^,^27^. Early studies on the REE showed 70% Nd removal in the Gironde estuary and 88% Nd removal in the Great Whale estuary^7^,^28^. A landmark study by Sholkovitz^29^ showed that 0.22-μm filtered REE concentrations also behave non-conservatively in the salinity gradient of the Amazon estuary. In the 0–6.6 salinity range, >90% of REE were removed from solution. This removal was attributed to coagulation of riverine colloids. Surprisingly, this study showed that all REE concentrations slightly increase again in the salinity range between 6.6 and 34.4. This was interpreted as being due to the release of REE from sediments and resuspended particles. An alternative explanation was the possibility of a certain amount of spatial and temporal (on a timescale of hours to days) heterogeneity in REE concentrations of the Amazon River plume as it mixes with seawater. Multiple studies have documented REE removal in estuaries and a REE concentration rebound at mid- to high-salinities^9^,^30^. Goldstein and Jacobson^31^ recognized early on that estuarine REE removal is important in balancing marine REE budgets. They also documented that the ɛNd of dissolved and suspended river loads can differ by up to four units, and that replacing one with the other in river ɛNd budgets is not trivial.

Here we present results obtained within the framework of the AMANDES 3 campaign to the Amazon estuary (Fig. 1) .We use filtration, ultrafiltration and Nd isotope analysis to reassess REE dynamics in the Amazon estuary salinity gradient and its impact on the river Nd flux to the Atlantic Ocean. We also use radium isotopes to provide constraints on the timescale of estuarine REE dynamics. We find that Nd release from Amazon river suspended sediments is rapid, and larger than the dissolved Nd river input to the Atlantic Ocean. We estimate that on a global scale the release process may dominate all other Nd inputs.

## Results

### REE behaviour in the Amazon estuary

**Figure 2:**
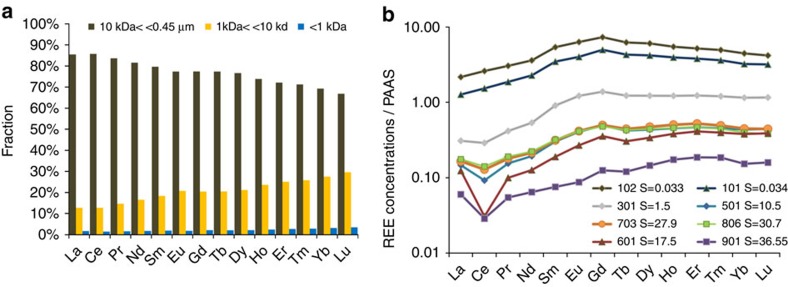
Dissolved REE speciation and total dissolved REE patterns across the Amazon estuary salinity gradient. (**a**) Operational REE speciation within the dissolved fraction (<0.45 μm) for the Amazon River endmember (this work). LREE are enriched in the coarse colloidal fraction (10 kDa<REE<0.45 μm) in comparison to the fine colloidal (1 kDa<REE<10 kDa) fraction and the ‘truly dissolved'<1 kDa fraction. (**b**) Post-Archean Australian shale (PAAS) normalized dissolved REE concentration along the Amazon estuary transect. The PAAS normalized Amazon River REE pattern loses its typical MREE enrichment^2^,^29^ by colloid coagulation, and evolves towards a HREE-enriched pattern that is more similar to Atlantic Ocean water.

The suspended particulate matter (SPM) Nd_SPM_ and dissolved Nd_<0.45 μm_ concentrations of the Amazon River endmember in April 2008 were 2,627 ng kg^−1^ and 123 ng kg^−1^, respectively (Supplementary Table 1). Within the dissolved phase, we find that coarse colloidal REE (defined here as 10 kDa<REE<0.45 μm) dominates REE speciation with Nd_<10 kDa_ of 22.5 ng kg^−1^ and Nd_<1k Da_ of 2.4 ng kg^−1^. Taken together, the filtration and ultrafiltration observations suggest that 95% of Amazon River Nd is in the particulate form when it enters the Amazon estuary. Of the remaining 5% of dissolved Nd_<0.45 μm_, 4.9% is present in the colloidal fraction >1 kDa (Fig. 2a). Barroux *et al.*^32^ studied the seasonal variations in dissolved (<0.2 μm) REE concentrations at Óbidos and reviewed all earlier published Amazon River REE data. Dissolved Nd_<0.2 μm_ varied between 47 ng kg^−1^ to 178 ng kg^−1^, during the low to high water stages from 2003 to 2005. Nd_<0.2 μm_ was shown to correlate significantly with the discharge of the Amazon River (eqs. (1), r2=0.64):


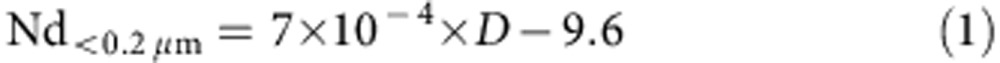


where *D* represents the discharge in m^3^ s^−1^. The Amazon discharge at the time of our observation in 2008 was 234,300 m^3^ s^−1^ (http://www.ore-hybam.org). Using this relationship we estimated a Nd_<0.2 μm_ concentration at Obidos of 154±40 ng kg^−1^ (1 s.d.), which is similar to our Nd_<0.45 μm_ observation of 123 ng kg^−1^. The TTO/TAS 44 Amazon river endmember samples 44 and 46 collected during the low water stage in December 1982 and reported in the study by Piepgras and Wasserburg^2^ displayed dissolved Nd concentrations of 53.9 and 48.5 ng kg^−1^, these values being also consistent with the seasonal dissolved Nd variation. Amazon River endmember normalized REE patterns in the different dissolved sub-phases show a light rare earth elements (LREE) enrichment in the coarse colloidal fraction compared with the fine colloidal fraction (defined here as 1 kDa<REE<10 kDa) and the truly dissolved phase (<1 kDa; Fig. 2a). This feature is in agreement with a laboratory mixing experiment using Connecticut River particles and water^19^. Within the colloidal fraction the main REE carriers are thought to be humic acids as well as iron and manganese oxyhydroxides whereas within the truly dissolved fraction, the main ligands that complex REE are carbonate ions and organic compounds that pass through the 1 kDa membrane such as fulvic acids^33^.

**Figure 3:**
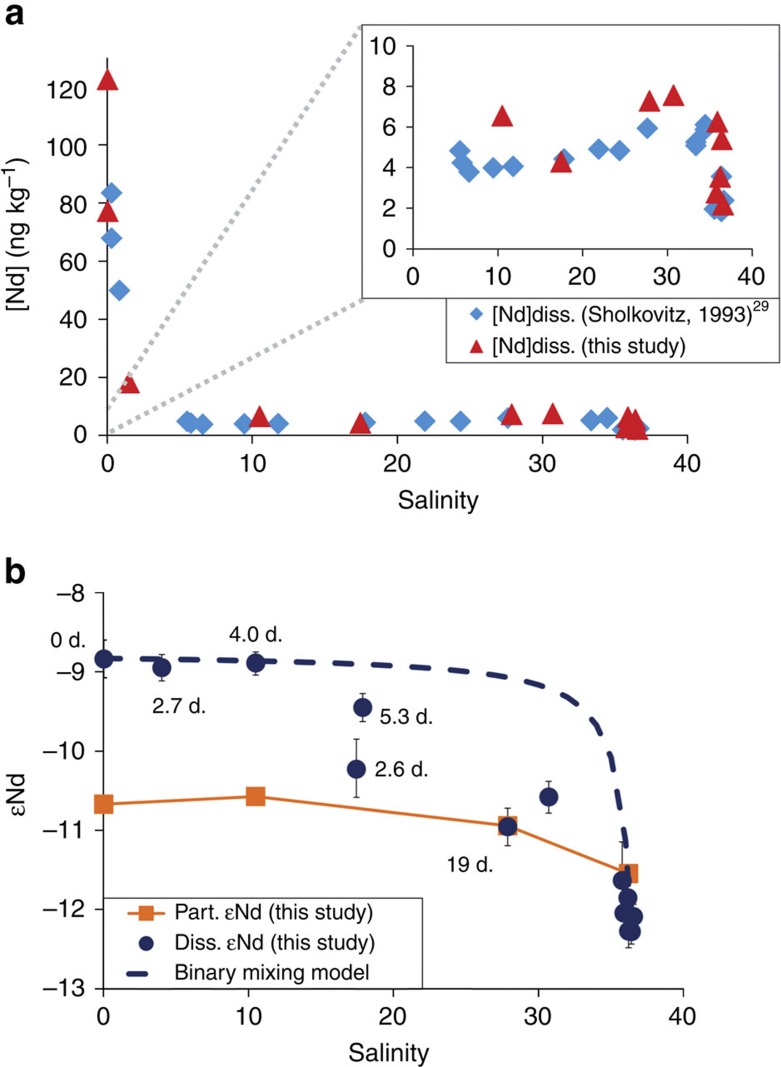
REE concentrations and Nd isotopic composition in the Amazon River estuary. (**a**) Amazon estuary Nd versus salinity gradient in the dissolved fraction in the study by Sholkovitz^29^ (blue diamonds), and this study (red triangles). (**b**) Amazon estuary ɛNd versus salinity in the dissolved (blue dots) and particulate (orange squares) fractions; the theoretical dissolved Nd conservative mixing curve is shown (blue-dashed line). The apparent radium ages estimated for the different water samples (expressed in days) are also reported.

Detailed observations of the Amazon Estuary obtained during the AmasSeds 1 campaign illustrated the so called colloidal flocculation for the REE^29^. The fraction of REEs that are removed from the dissolved phase in the salinity gradient must be calculated relative to the REE concentrations in the river endmember. Sholkovitz^29^ noted that the lowest AmasSeds 1 salinity samples (0.3) taken on 4–10 August 1989, and which have Nd concentrations of 83.5 and 67.9 ng kg^−1^, do not represent the true river endmember. Using equation (1) and the Amazon discharge at this time in 1989 (237,814 m^3^ sec^−1^) yields corresponding Nd concentration at Obidos of 157±40 ng kg^−1^. Figure 3a shows the Nd concentration gradient observed during both AmasSeds 1 and Amandes 3 campaign (this study and the study by Sholkovitz^29^). Similar to Sholkovitz observations, we find that total dissolved Nd<0.45 μm concentrations rapidly decrease in the low-salinity region (0–10) from 123 ng kg^−1^ to a minimum of 4.3 ng kg^−1^. Using the estimated river endmember of 157 ng kg^−1^ (1.1±0.3 μmol kg^−1^) and the observed Nd minima of 3.8 ng kg^−1^ (26.3 pmol kg^−1^) for the AmasSeds I campaign, both the Sholkovitz^29^ and this study show that >90% of dissolved Nd was removed from solution at the first stages of the Amazon Estuary. The dissolved Nd removal is strongest at a salinity of 17.5, a value which is higher than the AmasSeds 1 study (6.6) (Fig. 3a). This feature could be due to the use of different filter pore sizes in the two studies (that is, 0.45 μm here versus 0.22 μm during AmasSed) but more likely represents true natural variability, possibly related to seasonality (AmasSeds-1 in August 1989; Amandes-3 in April 2008). We observe that 98% of Amazon River Nd is in the >1 kDa colloidal fraction, and that the fine colloidal Nd_<10 kDa_ fraction gradually increases from the river endmember to the seawater endmember (that is, 18%, 53%, 73% and 90% for salinities 0.03, 1.5, 10.5 to 34.9 respectively, Supplementary Table 1). This trend confirms that large REE carrying colloids (between 10 kDa and 0.45 μm) flocculate out of solution along the estuarine mixing gradient as illustrated in Supplementary Fig. 1.

For salinities >17.5, dissolved REE concentrations slightly increase again, as also shown in previous studies^29^ (Fig. 3a). REE removal in the low-salinity zone is more pronounced for the LREE than the heavy rare earth elements (HREE), and REE release in the mid- to high-salinity zone is characterized by a slight preferential release of LREE to the dissolved Nd_<0.45 μm_ pool (Fig. 2b, Supplementary Fig. 2a,b). Consequently, the shale-normalized Amazon River REE pattern loses its typical middle rare earth element (MREE) enrichment^2^,^29^ by colloid coagulation, and evolves towards a HREE-enriched pattern that is more similar to Atlantic Ocean water (Fig. 2b).

### Determination of apparent water ages using Ra isotopes

Radium (Ra) has four radioactive isotopes that display different half-lives (^224^Ra, 3.66 days; ^223^Ra, 11.4 days; ^228^Ra, 5.75 years; ^226^Ra, 1,600 years). Ra isotopes can thus be used as clocks at different space and time scales. In freshwater, Ra is bound to suspended particles. Ra is then released into the dissolved phase once the freshwater enters in contact with seawater, due to the increased ionic strength^34^,^35^. The release of Ra isotopes to the dissolved phase allows us to use Ra isotopes as a tool to study the rate of water transport^36^,^37^. In this work, we determined apparent Ra ages for the water bodies investigated along the Amazon River plume, using the ^224^Ra/^223^Ra ratio as a clock. The ^224^Ra/^223^Ra ratio determined in each water sample is thus compared with the initial ratio determined at station AM3-0101 to derive an apparent age. The apparent ages of the water bodies increase as the Amazon plume moves towards offshore (Fig. 3b). We use these ages to estimate the rate of the Nd release that takes place within the Amazon plume that enters into the Atlantic Ocean.

### ɛNd behaviour in the Amazon estuary

The Nd isotopic composition of Amazon River SPM (>0.22 μm) has been reviewed elsewhere^38^, based on monthly ɛNd_SPM_ observations for Solimões and Madeira tributaries, which are the two main SPM carriers in the Amazon basin. It was observed that ɛNd of Solimoes SPM (−8.9 to −9.9) is slightly more radiogenic than ɛNd of Madeira SPM (−10.8 to −12.1). We calculate, by weighting the seasonal ɛNd variations by Solimoes and Madeira SPM concentrations and discharge that Amazon River SPM should have an annual mean ɛNd of −10.6. This estimate is based on samples collected >1,500 km from the estuary, yet in good agreement with the ɛNd of −10.7±−0.1 (>0.45 μm) analysed in this study for the estuarine Amazon River endmember (sample AM3-0102).

The dissolved phase ɛNd for the Amazon River endmember was determined during the Transient Tracers in the Ocean study (TTO/TAS) at stations 44 and 46, sampled in December 1982 in the mouth of the Amazon River. ɛNd of −8.4±0.5 and −9.2±0.4 were observed^39^. ɛNd of −8.9±0.5 was also reported in a subsequent study^2^. The dissolved phase ɛNd of −8.8±0.2 that we observe for the Amazon River endmember in this study (AM3-0102) is in excellent agreement with these earlier data. The offset that we observe between particulate and dissolved ɛNd in the Amazon River endmember suggests that a large fraction of the riverine particulate Nd is not exchangeable.

Figure 3b shows the evolution of ɛNd in the dissolved and particulate phases within the Amazon estuary salinity gradient. The apparent ages of the water bodies determined using radium isotopes—defined as the time elapsed since the river endmember entered in contact with seawater—are also reported for each water sample. Throughout the low-salinity region (0–17.5), as total dissolved Nd_<0.45 μm_ decreases from 123 to 6.6 ng kg^−1^, ɛNd_<0.45 μm_ remains constant at −8.9±0.1 (s.d., *n*=3). This suggests that ɛNd_<0.45 μm_ is uniform and exchangeable between the truly dissolved and colloidal pools. At salinities higher than 17.5, when Nd_<0.45 μm_ reaches its minimum of 4.3 ng kg^−1^ and then increases to 6–7 ng kg^−1^, we observe a gradual decrease in ɛNd_<0.45 μm_ down to −11.0. This latter pattern is observed in water bodies displaying an apparent radium age of 19 days. Although the Atlantic Ocean endmember that mixes with Amazon River water has a low ɛNd_<0.45 μm_ of about −12.1, conservative mixing of Amazon and marine Nd alone is not sufficient to explain the ɛNd_<0.45 μm_ gradient in the Amazon estuary, let alone the increase in total dissolved Nd in the mid-salinity region. The lower ɛNd of the Nd_SPM_ released into the dissolved phase also indicates that it unlikely originates from desorption of exchangeable Nd_SPM_ bound to recently coagulated colloids or particulate organic carbon in SPM, as these should have an ɛNd close to dissolved Nd (−8.9). We observed that Amazon estuary SPM has an average ɛNd of −10.7. This pattern therefore suggests that the release of a small fraction of particulate lithogenic Nd is responsible for the decrease in ɛNd of total dissolved Nd at salinity>17.5.

## Discussion

It is of interest to discuss whether the mechanism of Nd_SPM_ release is desorption of SPM-bound Nd, or dissolution of Nd-containing SPM solid phases. Two lines of evidence argue for a dissolution mechanism. First, if Nd_SPM_ on mixing with seawater were to be desorbed from binding sites on particulate organic matter or Fe and Mn oxyhydroxide surfaces, the Nd would have to be per definition exchangeable. Exchangeable Nd_SPM_ would then have to have a uniform ɛNd between the river water dissolved and releasable Nd_SPM_ pools. Uniform ɛNd is not what we observe. Second, we see a decoupling between the release of Ra_SPM_ and Nd_SPM_ along the salinity gradient, Ra being released in the 0–5 salinity range, whereas Nd is released at higher salinity (ca. 17.5). This suggests that two different mechanisms are at play. The rapid release of Ra when the freshwater enters in contact with seawater is known to be associated with the desorption of Ra from SPM^34^,^36^. The release of Nd_SPM_ at higher salinity may suggest a slower process assuming that the chemical reactions that promote such release have also started when the freshwater enters in contact with seawater. The observed release of Nd_SPM_ in the salinity gradient may thus be mostly driven by dissolution processes, rather than by desorption.

We apply an isotope mass balance to quantify the fraction of Nd that is released from SPM along the estuarine gradient (see Methods). Three major dissolved Nd fractions are defined in the estuarine gradient relative to total dissolved Nd: the remaining Amazon River fraction, *f*_Ama_; the fraction from the Atlantic seawater endmember, *f*_Atl_; and the fraction released from SPM, *f*_SPM_. We assume that the three dissolved Nd fractions have corresponding ɛNd_Ama_, ɛNd_Atl_ and ɛNd_SPM_ of −8.8, −12.1 and −10.7, respectively. The fraction of Nd released from sediments becomes significant at a salinity of 17.5. We calculate that in the salinity ranges of 0–10, 17.5 and 28–30 (corresponding to our samples) ∼0, 1.5 and 4.9±1.2 (s.d.) ng of Nd per kg of estuarine water has been released from SPM, respectively (Supplementary Table 2). The quoted uncertainty (s.d.) is based on averaging over two samples that define the 28–30 salinity range. To evaluate the magnitude of the 4.9 ng kg^−1^ Nd released at salinity 28–30, we need to correct this value for dilution with seawater. The resulting concentration of 24.7±6.0 ng kg^−1^ of Nd_SPM_ released at ‘0' salinity can now be compared with the particulate Nd_SPM_ concentration in the Amazon River in April 2008 (that is, 2,600±260 ng kg^−1^, rounded off to two significant figures). We calculate that approximately 0.94%±0.25 % (propagated s.d.) of particulate Amazon River Nd_SPM_ was released to the dissolved Nd pool in the estuary.

The Amazon River-dissolved Nd flux to the Atlantic Ocean can be summarized based on the detailed seasonal REE observations by Barroux *et al.*^32^ and assuming that our observed Nd removal and Nd release are constant throughout the year: 93% of the annual 607 Mg per year Nd_<0.45 μm_ flux^32^ is removed by colloid coagulation in the low-salinity zone, transferring the remaining 7%, that is, 42 Mg per year to the Atlantic Ocean. Here we estimate that 0.94% of the annual 19,800 Mg per year Nd_SPM_ flux (based on a Nd_SPM_ of 33 mg kg^−1^ (8) and Amazon sediment discharge of 6 10^14^ g y^−1^ (ref. 40)) is released from SPM in the mid- to high-salinity zone, that is, 186 Mg per year. The Nd release from SPM is therefore approximately four times larger than the dissolved Nd river flux to the Atlantic Ocean. The typical MREE enriched pattern of the Amazon River-dissolved pool is thus near-quantitatively removed to SPM and sediments in the salinity gradient and replaced by a HREE-enriched pattern that derives from the partial dissolution of SPM and approaches that of Atlantic Ocean waters (Fig. 2b).

**Figure 4:**
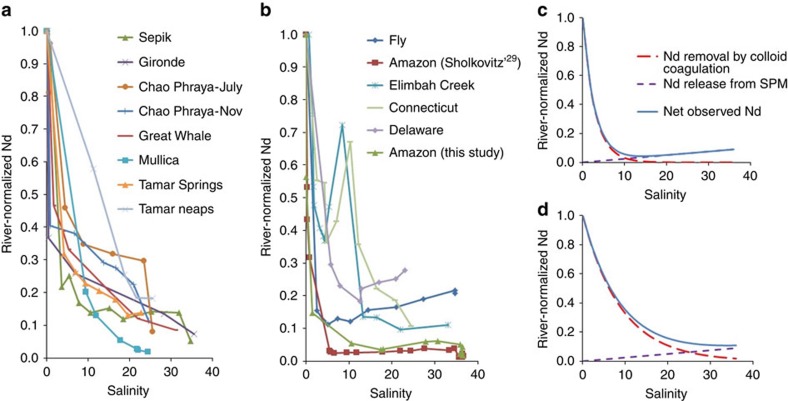
Overview of published estuarine dissolved Nd observations. In this overview of published estuarine dissolved Nd observations^7^,^9^,^28^,^29^,^30^,^51^,^52^,^53^,^54^,^55^ all Nd concentrations are normalized to that of the corresponding river endmember. (**a**) estuaries that show a gradual drop in Nd concentrations, (**b**) estuaries that show both an initial drop (removal) followed by a rebound (release) in Nd concentrations, (**c**) scenario illustrating simultaneous fast Nd removal and slow Nd release, (**d**) scenario illustrating simultaneous slow Nd removal and slow Nd release. The removal and release scenarios in **c** and **d** correspond to the observed trends in **a** and **b**, respectively.

In the following we compare our observations to published estuarine REE studies and examine the implications of Nd_SPM_ release on the transfer of Nd globally. Table 1 summarizes published observations of estuarine dissolved Nd dynamics, pH, SPM and dissolved organic carbon (DOC). Figure 4a,b shows all published estuarine Nd_<0.45 μm_ concentration trends, normalized to the corresponding river water Nd_<0.45 μm_. All studies show a pronounced removal of Nd_<0.45 μm_ in the low-salinity range (0–10) that is thought to represent REE-carrying colloid coagulation^9^,^20^,^31^. Two types of patterns can be observed in the mid to high salinity range (10–36): a gradual decrease in Nd_<0.45 μm_ to concentrations that are close to the seawater endmember (Fig. 4a), or a decrease in Nd_<0.45 μm_ followed by a more or less pronounced increase in Nd_<0.45 μm_ (Fig. 4b). On the basis of our ɛNd observations in the Amazon estuary, we argued above that the Nd_<0.45 μm_ rebound is due to dissolutive Nd release from SPM. Apart from a dilution due to mixing, Amazon estuary Nd dynamics appear therefore to be controlled by two competing mechanisms: a decrease in Nd_<0.45 μm_ due to colloid coagulation, and an increase in Nd_<0.45 μm_ due to SPM dissolution. The relative magnitude of Nd removal and release likely depend on SPM mineralogy, river pH, DOC and major elements, amongst others. We illustrate in Fig. 4c,d how natural variations in Nd removal and release can result in observed estuarine Nd trends that cover most published observations. For example, rapid and pronounced Nd removal (Fly and Amazon) is more likely to reveal the dissolutive Nd_SPM_ release at mid-high salinities (Fig. 4c). Slower Nd removal in combination with identical Nd_SPM_ release may visually hide the Nd rebound in observations (Fig. 4d). In this latter case Nd isotopes may be useful to quantitatively discern the two processes.

Goldstein and Jacobson^31^ examined early studies on estuarine Nd_<0.45 μm_ removal to identify the controlling river chemistry parameters. They postulated that low pH, high Nd rivers should show more Nd removal. The two available studies on the Gironde (low Nd, 7) and Great Whale (high Nd^28^), however, showed similar Nd removal of ∼70%. This removal factor has subsequently become the benchmark value in marine Nd models (14,15,16). We calculated maximum Nd_<0.45 μm_ removal for the 15 estuarine transects (Table 1). The percentage Nd_<0.45 μm_ removal data are normally distributed, with a mean and s.d. of 71±16%. Nd removal is not correlated with pH (*r*^2^=0.03), or with DOC (*r*^2^=0.02), suggesting that the historically used nominal 70% Nd removal value^31^ can be used for rivers globally. Future work should assess Nd_SPM_ release % for a large number of estuaries by examining dissolved and SPM Nd concentration and ɛNd gradients.

The implication of our findings for the global marine Nd budget can at this point only be assessed from our single Amazon estuary observation of Nd_SPM_ release. We use a global riverine sediment flux to the oceans of 1.85±0.16 10^16^ g per year^41^,^42^,^43^,^44^. Mean river Nd_SPM_ concentrations are 33±12 μg g^−1^ (ref. 21) and we observe in this study that 0.94±0.25% of river Nd_SPM_ is released on mixing with seawater. We thus estimate that globally 5,700±2,600 Mg of Nd (s.d. by error propagation; rounded off to two significant figures) is released annually to coastal marine waters. The estimated range of 3,100–8,300 Mg per year is 6–17 times larger than the 500 Mg per year dissolved river-Nd load^31^ (taking into account the globally observed 70% estuarine removal). It is also 8–21 times larger than the atmospheric dust Nd flux, assuming 2% dust dissolution, of 400 Mg per year 14. The Nd release range overlaps with the model estimated missing Nd flux of 5,500–11,000 Mg per year, but may still be up to 3.5 times smaller, based on the lower observed (3,100) and upper modelled (11,000) extremes.

Using radium isotopes to constrain the timescale of the chemical reactions taking place on Amazon River water mixing with seawater, we can further argue that coagulation and Nd-release processes occur rapidly, within ∼19 days (3±1 weeks). The magnitude and timing of Nd release from sediments observed in this study is therefore also coherent with batch experiments where basaltic lithogenic particles of diverse origins were incubated with seawater^17^.

In summary, our observations suggest that dissolved REE have little influence on the Nd isotopic composition of the Amazon plume and the Atlantic Ocean. Rather, the release of REE from suspended river sediments dominates REE concentrations and ɛNd of the Amazon River plume over the northeast Brazilian shelf. The large amount of Nd released from lithogenic suspended sediments carried by the Amazon River over limited space and time scales underlines that river sediments may contribute significantly to the global marine dissolved Nd budget and possibly to that of other chemical elements.

## Methods

### Sample collection and treatment

Samples were obtained on the RV/ANTEA in April 2008 (AMANDES 3 campaign) and were collected in 8-l Niskin bottles mounted on a 12-bottle CTD-equipped rosette. For Nd isotopic analysis, 10 l were filtered on board with 0.45-μm polyethersulfone (PES) Supor filters. Eight-litre aliquots (out of 10 l) were immediately acidified to pH 3.5 using double-distilled 6 M HCl and 7.5 l was preconcentrated onboard using two C18 SepPak cartridges loaded with a strong REE complexant (HDEHP/H_2_MEHP). The remaining 0.5 l acidified sample was conserved for total dissolved REE analysis. Additional onboard 10- and 1-kDa ultrafiltration, using Millipore ultrafiltration cartridges, was done on 2-l unacidified aliquots to observe in detail the REE features of the coarse REE-colloidal (10 kDa<REE<0.45 μm), fine REE-colloidal (<10 kDa; >1 kDa) and truly dissolved fractions (<1 kDa). These ultrafiltered samples were subsequently acidified using double-distilled 6 M HCl.

Back at the land-based LEGOS laboratory, the REE preconcentrated on C18 cartridges were eluted using 6 M HCl, evaporated and redissolved in 1.5 ml of 1 M HCl. Nd separation was achieved by a two-step chromatography protocol using cationic AG50 X8 and Ln-SPEC resins. After evaporation to dryness and dissolution again in 2 M HCl, C18 eluate was loaded on a cation-exchange column (0.6 cm in diameter, 4.8 cm in height) packed with Biorad AG50W-X8 (200–400 mesh) resin to extract the REE from the remaining matrix using HCl and HNO_3_. The REE were then eluted with 6 ml of 6 M HCl. This solution was evaporated and redissolved in 0.3 ml of 0.2 M HCl for the final extraction of Nd using an anion-exchange column (0.4 cm in diameter, 4 cm in height) packed with 0.5 ml of Ln-Spec resin. A final elution using 2.5 ml of 0.2 M HCl allowed recovering the neodymium.

### REE and ɛNd analysis

Nd isotope measurements were made on a Thermo Finnigan MAT 261 at the Observatoire Midi-Pyrénées in dynamic mode, correcting for instrumental mass bias using a ^146^Nd/^144^Nd ratio of 0.7219. Repeated analysis of the La Jolla standard gave a ^143^Nd/^142^Nd ratio of 0.511843±0.000020 (2 s.d., *n*=49) in agreement with the recommended value of 0.511858^45^. Blank contributions to the Nd isotopic measurement were on average <3% of the total signal. Suspended particles (>0.45) samples were analysed at the Ifremer laboratory of Brest following the method described in ref. 46. The Nd isotope signature is defined as ɛNd, the ratio of radiogenic ^143^Nd over stable ^144^Nd, normalized to CHUR (Chondritic Uniform Reservoir, ^143^Nd/^144^Nd=0.512638) on the parts per ten-thousand scale (equation (2)); which represents the present-day average earth value^47^:


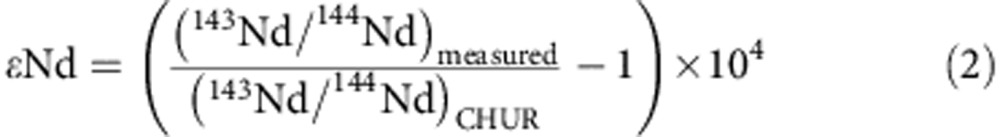


A new multiple isotope dilution—sector field ICP-MS method was used to quantify REE concentrations with a precision <2% for most REE, except La and Ce<5%^28^. For REE concentration analysis 500-ml sample aliquots were taken from the 10-l <0.45-μm filtrates and 2-l ultrafiltration permeates. The samples were then spiked with a mix of 10 artificially enriched REE isotopes and subsequently preconcentrated by iron co-precipitation followed by AG1-X8 and AG50-X8 ion chromatography. Samples were analysed using a sector field ICP-MS (Thermo Scientific Element-XR) at Observatoire Midi-Pyrénées. Details on spiking, separation and analysis procedures can be found in ref. 48.

### Radium analysis

In parallel, Ra isotopes were analysed in water samples collected in the Amazon plume within the salinity gradient. Briefly, large volumes of water (up to 200 l) were collected and passed through cartridges filled with Mn fibres. ^224^Ra (*T*_1/2_=3.66 days) and ^223^Ra (*T*_1/2_=11.4 days) activities were determined on the ship using a Radium Delayed Coincidence Counter^49^,^50^. Following Moore^35^, we used the ^224^Ra/^223^Ra ratios to provide apparent ages for the water bodies sampled along the Amazon plume.

### ɛNd mass balance calculation

The Nd isotope mass balance considers three dissolved Nd fractions: the remaining Amazon River fraction, *f*_Ama_, the fraction from the Atlantic seawater endmember, *f*_Atl_, and the fraction released from suspended sediments, *f*_sed_. We do not take into account coagulated Nd in this mass balance as it has been transferred to the particulate phase. If a subfraction of coagulated Nd were to desorb and regain the dissolved solution phase, it is automatically included in *f*_Ama_. We assume that Nd in the *f*_Atl_ fraction mixes conservatively in the salinity gradient, as it is predominantly bound to <10-kDa colloids. We assume that the three dissolved Nd fractions, *f*_Ama_, *f*_Atl_ and *f*_sed_, have corresponding ɛNd_Ama_, ɛNd_Atl_ and ɛNd_sed_ of −8.8, −12.1 and −10.7, respectively. Calculated fractions that were not significantly different from 0 and 1 have been reset to 0 and 1, respectively. The mass balance was not calculated for 3 deep samples of high Nd concentrations and slightly lower ɛNd than the highest salinity sample taken as Atlantic endmember (Supplementary Discussion). As the ɛNd of the three endmembers span a relatively narrow range, the uncertainties on the independent variables, *f*, are relatively large. We therefore average results over three salinity ranges in the gradient (0–10, 17.5 and 28–30 salinity, see Supplementary Table 2).

## Additional information

**How to cite this article:** Rousseau, T. C. C. *et al.* Rapid neodymium release to marine waters from lithogenic sediments in the Amazon estuary. *Nat. Commun.* 6:7592 doi: 10.1038/ncomms8592 (2015).

## Supplementary Material

Supplementary Information Supplementary Figures 1-2, Supplementary Tables 1-2, Supplementary Discussion and Supplementary References

## Figures and Tables

**Figure 1:**
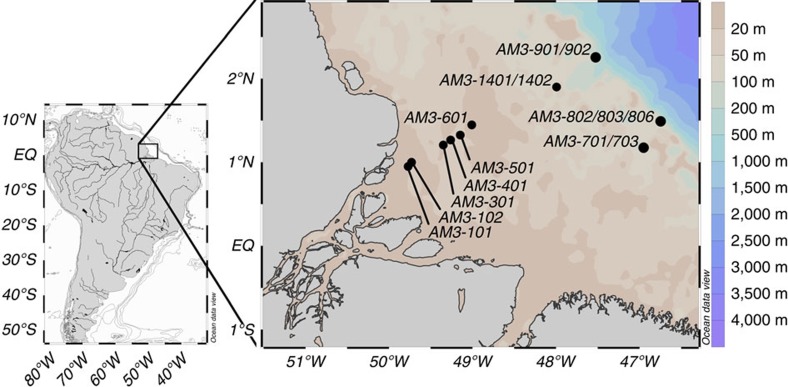
Sampling stations of the AMANDES 3 cruise in the Amazon River estuary. The AMANDES 3 campaign was realized between the 6 April 2008 and the 18 April 2008 on the R/V ANTEA in the Amazon estuary shelf as part of the GEOTRACES programme. Surface samples were collected within the salinity gradient and depth profiles were obtained near the break of the shelf.

**Table 1:** Summary of published studies on estuarine Nd dynamics.

**River**	**Dissolved Nd ng kg**^**−1**^	**pH**	**DOC mg l**^**−1**^	**Nd removal %**	**Reference**
Amazon	157	6.9	5.1	97	29
Amazon	123	6.9	5.1	93	This study
Fly	26	7.8	3.2	87	30
Sepik	36	7.3	3.4	81	30
Gironde	38	7.7	3.1	68	7
Luce	53	4.5	9.0	73	53
Elimbah Creek	103	6.1		79	52
Chao Phraya river—July	14	7.0		49	51
Chao Phraya river—November	14	7.0		58	51
Great Whale	167	6.8	5.3	66	28
Vora Creek	177	4.4	12.8		52
Vora Creek	165	4.6	9.8		54
Connecticut	357	7.0	3.2	56	55
Delaware	26	7.5	2.6	70	55
Mullica	412	4.6		81	55
Tamar Springs	37	7.25		65	55
Tamar neaps	33	7.25		40	55

DOC, dissolved organic carbon; Nd, neodymium

River endmember dissolved Nd is shown, and maximum Nd removal was calculated as the strongest drop in Nd along the estuarine transect, after correcting for mixing with seawater. Mean Nd removal is 71±16% (s.d.).
