# Supplementary material for: Rapid neodymium release to marine waters from lithogenic sediments in the Amazon estuary
Source: Nat Commun. 2015 Jul 9;6:7592. doi: 10.1038/ncomms8592 (PMC4510642; doi:10.1038/ncomms8592)
Supplement: Supplementary Information — Supplementary Figures 1-2, Supplementary Tables 1-2, Supplementary Discussion and Supplementary References [file ncomms8592-s1.pdf]

Supplementary Figure 1: Evolution of the coarse colloidal REE fraction in the Amazon estuary salinity gradient.

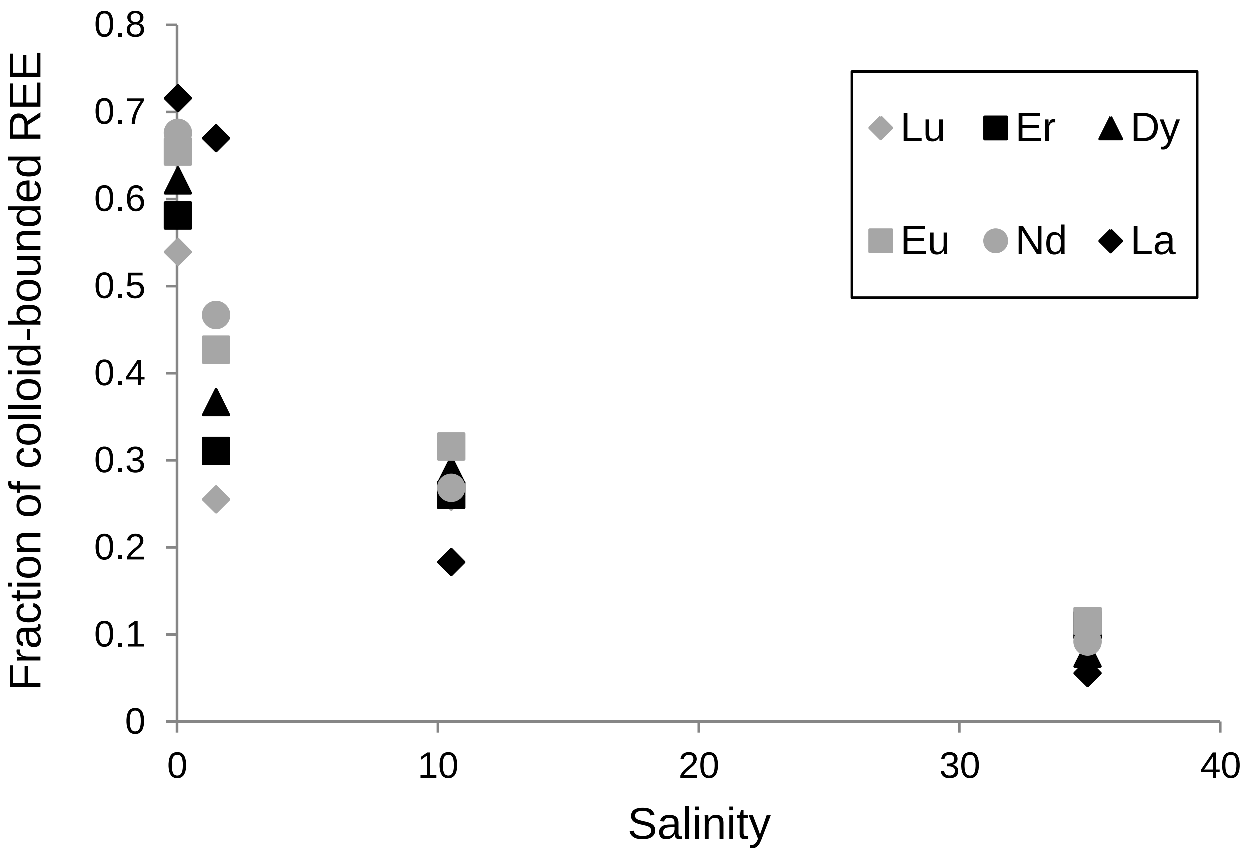

Colloidal REE concentrations in the 10kDa>REE>0.45µm pore size fraction normalized by their respective concentrations in the total dissolved fraction (<0.45µm) (as the colloidal fraction was not analyzed for the estuarine seawater end-member AM3-0803, sample AM2-09 of salinity 34.9 collected in February 2008 offshore of the Guyana margin is reported for comparison).

Supplementary Figure 2 Dissolved REE patterns within the amazon estuary

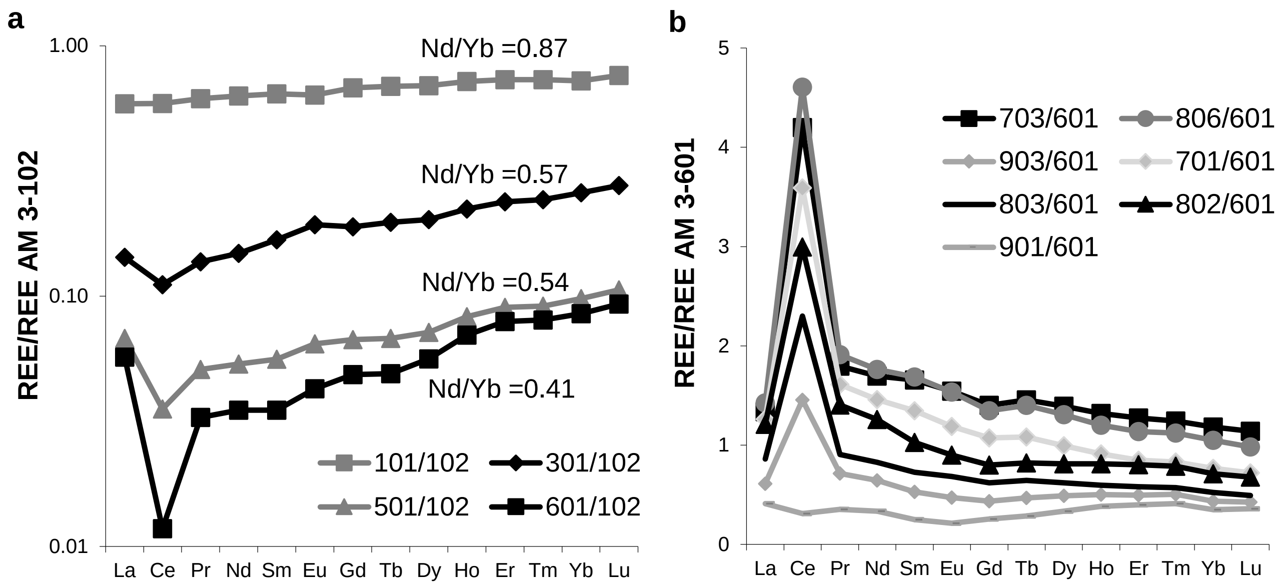

Amazon estuary REE concentrations (<0.45μm) a) in the concentration drop zone normalized to the Amazon end-member station (AM3-102, salinity 0.3). Stations AM3-0101, AM3-0301, AM3-0501, AM3-0601 have respective salinities of 0.034, 1.50, 10.51 and 17.45. Note logarithmic scale on y-axis. Similarly to Sholkovitz (1993)[1] we observe a preferential LREE over HREE removal with salinity increase and a Nd/Yb ratio evolving from 0.87 to 0.57, 0.54 to 0.41 (for AM3-0101, AM3-0301, AM3-0501 and AM3-0601 samples respectively); Lu is thus the least affected by removal. b) within the mid to high salinities region (after the concentrations drop zone) normalized to the Amazon estuary station AM3-601, (salinity 17.45, strongest REE removal). Stations AM3-0703, AM3-0806, AM3-0903, AM3-0701, AM3-0803, AM3-0801, and AM3-0901 have respective salinities of 27.88, 30.70, 35.77, 35.89, 36.20, 36.40 and 35.77. Note linear scale on y-axis. All samples with salinity >17.5 are characterized by LREE enrichment compared to AM3-0601 suggesting the REE release process from sediment favors LREE over HREE.

Supplementary Table 1: Hydrological and geochemical data. Long., Lat., Temp., Sal., Cond. and Oxy. stands for Longitude, latitude, Temperature, Salinity, conductivity and dissolved Oxygen. REE concentration are expressed in ng kg^{-1}.

Station	Date	Long.	Latit.	Depth	Temp.	Sal.	pH	Cond. (mS)	Oxy. ml/l	εNd	2SE		La	Ce	Pr	Nd	Sm	Eu	Gd	Tb	Dy	Ho	Er	Tm	Yb	Lu
AM3-0101	10/04/2008	-49.76	0.96	6.9	27.75	0.034		$85,3 .10^{-3}$	1.89			<0.45 µm	48.63	121.97	16.55	77.23	19.32	4.34	23.17	3.35	19.66	3.91	10.83	1.47	9.11	1.39
AM3-0102	10/04/2008	-49.74	0.98	6.9	27.75	0.033	7.035	$49,6 .10^{-3}$	2.13	-8.8	0.2	<0.45 µm	82.86	207.45	26.96	122.63	30.04	6.66	34.13	4.86	28.36	5.42	14.78	2.01	12.59	1.83
										-10.7	0.1	>0.45 µm				2627										
												10kdP	12.00	29.46	4.39	22.51	6.10	1.54	7.70	1.10	6.61	1.42	4.11	0.58	3.86	0.61
												1kdP	1.43	2.97	0.43	2.18	0.58	0.12	0.72	0.10	0.61	0.13	0.40	0.06	0.40	0.06
AM3-03	10/04/2008	-49.36	1.21	7	27.85	1.5	7.29	3,41	2.37			<0.45 µm	11.83	23.00	3.69	18.18	5.04	1.32	6.46	0.96	5.74	1.21	3.51	0.49	3.25	0.51
												10kdP	3.90	9.78	1.81	9.70	2.88	0.75	3.82	0.58	3.63	0.80	2.42	0.35	2.38	0.38
AM3-04	10/04/2008	-49.26	1.26	7.2	27.58	4.03	7.32	7,75	2.35	-9	0.2	<0.45 µm	*													
												10kdP	5.37	9.44	1.69	8.75	2.40	0.64	3.31	0.50	2.96	0.66	1.94	0.27	1.84	0.29
AM3-05	10/04/2008	-49.15	1.34	3.1	27.67	10.51	7.70	17,83	3.25	-8.9	0.1	<0.45 µm	5.61	7.35	1.37	6.56	1.68	0.44	2.29	0.33	2.03	0.45	1.33	0.18	1.23	0.19
										-10.6	0.1	>0.45 µm														
												10kdP	4.58	5.61	0.98	4.80	1.17	0.30	1.62	0.23	1.45	0.32	0.98	0.13	0.90	0.14
AM3-06	10/04/2008	-49.02	1.45	4.3	27.6	17.45	7.889	-	3.16	-10.2	0.4	<0.45 µm	4.72	2.44	0.88	4.30	1.05	0.29	1.66	0.24	1.59	0.38	1.17	0.16	1.07	0.17
AM3-0701	11/04/2008	-46.97	1.17	43.1	27.61	35.89		-	3.1	-12.1	0.2	<0.45 µm	5.96	8.77	1.42	6.27	1.42	0.35	1.78	0.26	1.58	0.34	0.99	0.13	0.82	0.12
AM3-0703				5	28.29	27.88		-	3.72	-11	0.2	<0.45 µm	6.32	10.25	1.59	7.29	1.74	0.45	2.32	0.35	2.22	0.50	1.49	0.20	1.26	0.19
										-10.9	0.1	>0.45 µm														
AM3-0802	11/04/2008	-46.73	1.49	93.1	25.13	36.4		-	3.38	-12.3	0.2	<0.45 µm	5.72	7.31	1.24	5.40	1.08	0.26	1.32	0.20	1.29	0.31	0.94	0.13	0.76	0.12
AM3-0803				29	27.72	36.2	8.31	-	3.52	-12.3	0.2	<0.45 µm	4.06	5.61	0.80	3.55	0.76	0.20	1.03	0.15	0.99	0.23	0.68	0.09	0.56	0.08

									-11.5	0.1	>0.45 μm														
AM3-0806				4	27.88	30.7	-	3.67	-10.6	0.2	<0.45 μm	6.72	11.24	1.69	7.57	1.77	0.45	2.23	0.33	2.08	0.45	1.33	0.18	6.72	11.24
AM3-0903	12/04/2008	-47.50	2.26	9.2	27.98	35.77	-	3.5	-11.6	0.5	<0.45 μm	2.88	3.55	0.63	2.77	0.55	0.14	0.72	0.11	0.78	0.19	0.58	0.08	2.88	3.55
AM3-0901				88.4	24.76	36.55	-	3.39	-12.1	0.2	<0.45 μm	2.30	2.29	0.48	2.19	0.42	0.09	0.58	0.09	0.68	0.17	0.53	0.08	2.30	2.29
AM3-14	12/04/2008	-48.00	1.91	51.4	27.01	36.16	-	3.07	-11.9	0.3	<0.45 μm														
AM3-14				4	28.12	17.88	-	4.41	-9.5	0.2	<0.45 μm														
AM2-09	22/01/2008	-51.47	6.57	3229	2.460	34.91					<0.45 μm	4.15	0.97	0.71	3.25	0.66	0.20	0.94	0.14	1.07	0.28	0.96	0.14	0.91	0.16
											10kdP	4.02	1.29	0.67	3.03	0.59	0.18	0.87	0.14	1.01	0.26	0.88	0.13	0.84	0.15

*Sample lost during REE analysis

Supplementary Table 2: Nd isotope mass balance results for estimating dissolved Nd fractions in the Amazon River salinity gradient.

	Salinity	εNd	$Nd_{<0.45\mu m}$ ng kg^{-1}	fNd_{Atl}	fNd_{Ama}	fNd_{SPM}
AM3-0102	0.03	-8.84	122.6	0	1	0
AM3-0501	10.51	-8.89	6.56	0	1	0
AM3-0601	17.45	-10.23	4.30	0.24	0.43	0.33
AM3-0703/0806	29.29	-10.77	7.41	0.24 ±0.01	0.13 ±0.16	0.63 ± 0.17
AM3-0903	35.77	-11.63	2.77	0.77	0.07	0.16
AM3-0901	36.55	-12.1	2.19	1	0	0

Supplementary Discussion

Possible evidence for boundary exchange on the Brazilian shelf

In the >35 salinity range there is also evidence for a contribution of sediment sourced Nd, as Nd concentrations of 3.6 to 6.3 ng kg^{-1} are elevated over those of the reference Atlantic Ocean end-member (2.2 ng kg^{-1}). Samples AM3-0701, AM3-0802 and AM3-0803 were collected near the break of the continental slope at bottom depth (43, 29 and 93m) where the Amazon freshwater has no influence. During the AmasSeds 1 study, Sholkovitz (1993) also observed deep samples with elevated REE concentrations and attributed it to a possible release from bottom sediments. We therefore suspect that Nd release from sediments is indeed occurring on the Brazilian shelf, as more broadly hypothesized by Lacan and Jeandel (2005)[2] and further modelled by Arsouze et al, (2009)[3]. We note that we have no observations of εNd in Brazilian shelf sediments or SPM from rivers reaching the Atlantic Ocean south of the Amazon estuary. These would be needed to understand Nd boundary exchange over the Brazilian shelf in more detail.

Supplementary References

1 Sholkovitz, E. R. The geochemistry of rare earth elements in the Amazon River estuary. *Geochimica et Cosmochimica Acta* **57**, 2181-2190 (1993).

2 Lacan, F. & Jeandel, C. Neodymium isotopes as a new tool for quantifying exchange fluxes at the continent-ocean interface. *Earth and Planetary Science Letters* **232**, 245-257 (2005).

3 Arsouze, T., Dutay, J. C., Lacan, F. & Jeandel, C. Reconstructing the Nd oceanic cycle using a coupled dynamical - biogeochemical model. *Biogeosciences* **6**, 2829-2846 (2009).
